# α_4_β_1_ and α_M_β_2_ Integrin Expression and Pro-Proliferative Properties of Eosinophil Subtypes in Asthma

**DOI:** 10.3390/jpm11090829

**Published:** 2021-08-24

**Authors:** Egle Jurkeviciute, Andrius Januskevicius, Airidas Rimkunas, Jolita Palacionyte, Kestutis Malakauskas

**Affiliations:** 1 Laboratory of Pulmonology, Department of Pulmonology, Lithuanian University of Health Sciences, LT-44307 Kaunas, Lithuania; andrius.januskevicius@lsmuni.lt (A.J.); airidas.rimkunas@lsmuni.lt (A.R.); kestutis.malakauskas@lsmuni.lt (K.M.); 2 Department of Pulmonology, Lithuanian University of Health Sciences, LT-44307 Kaunas, Lithuania; jolita.palacionyte@lsmuni.lt

**Keywords:** eosinophil subtypes, lung-resident eosinophils, inflammatory eosinophils, asthma, integrins, airway smooth muscle, apoptosis, proliferation

## Abstract

Eosinophilic inflammation is one of the main pathophysiological features in asthma. Two subtypes of eosinophils exist in the lung and systemic circulation: lung-resident eosinophils (rEOS) and inflammatory eosinophils (iEOS). We evaluated the expression of α_4_β_1_ and α_M_β_2_ integrins of eosinophil subtypes and their influence on airway smooth muscle (ASM) cell proliferation and viability in asthma. We included 16 severe non-allergic eosinophilic asthma (SNEA) patients, 13 steroid-free, non-severe allergic asthma (AA) patients, and 12 healthy control subjects (HS). For AA patients, a bronchial allergen challenge with *Dermatophagoides pteronyssinus* was performed. The eosinophil subtypes were distinguished using magnetic bead-labeled antibodies against surface CD62L, and individual combined cell cultures were prepared with ASM cells. The integrins gene expression was analyzed by a quantitative real-time polymerase chain reaction. Proliferation was assessed by the Alamar blue assay, and viability by annexin V and propidium iodide staining. rEOS-like cells were characterized by the relatively higher gene expression of the β_1_ integrin subunit, whereas iEOS-like cells were characterized by the α_M_ and β_2_ integrin subunits. The inclusion of either eosinophil subtypes in co-culture significantly increased the proliferation of ASM cells, and the effect of rEOS-like cells was stronger than iEOS-like cells (*p* < 0.05). Furthermore, rEOS-like cells had a more pronounced effect on reducing ASM cell apoptosis compared to that of iEOS-like cells (*p* < 0.05). Lastly, the bronchial allergen challenge significantly enhanced only the iEOS-like cells’ effect on ASM cell proliferation and viability in AA patients (*p* < 0.05). These findings highlight the different expression of α_4_β_1_ and α_M_β_2_ integrins on distinct eosinophil subtypes in asthma. Therefore, rEOS-like cells have a stronger effect in stimulating ASM cell proliferation and viability; however, contact with specific allergens mainly enhances pro-proliferative iEOS-like cell properties.

## 1. Introduction

Asthma is a chronic inflammatory lung disease that globally affects more than 300 million people. It is characterized by activated inflammatory cells, increased inflammatory mediators, airway hyperresponsiveness, intermittent or fixed airway obstruction, and airway remodeling [1]. Asthma is an incurable disease; thus, only the symptoms and severity of the disease can be controlled through the use of appropriate medication and avoiding irritants. The majority of the symptoms occur due to abnormal chronic airway inflammation, and eosinophils are the most involved effector cells. Eosinophilic inflammation is associated with disturbed airway homeostasis caused by the abundant production of various chemokines, cytokines, lipid mediators, and growth factors [2].

One of the causes of asthma complexity might reside in the existence of two distinct eosinophil subtypes that differ according to their role in asthma pathogenesis. One subtype is identified as lung-resident eosinophils (rEOS), and the other is inflammatory eosinophils (iEOS). Moreover, rEOS dwell in lung tissue throughout life in stable quantities, where they regulate local immunity. Meanwhile, blood iEOS mainly penetrate the airways in response to an environmental stimulus, such as an allergen, and depart along with bronchial secretions, and their cell numbers increase after allergen-induced airway inflammation [3]. Similar markers expressing eosinophils can be found in circulation and are called rEOS-like cells and iEOS-like cells. Furthermore, the subtypes of blood-circulating eosinophils are specific for asthma phenotypes: iEOS-like cells predominate in allergic asthma (AA), and rEOS-like cells in severe non-allergic eosinophilic asthma (SNEA) [4].

Airway remodeling is closely related to increased ASM mass due to impaired ASM cell proliferation, resulting in increased cell numbers and extracellular matrix secretion [5]. Eosinophils are a significant source of pro-proliferative mediators. Mediators secreted by eosinophils, such as tumor necrosis factor-alpha (TNF-α), transforming growth factor-beta (TGF-β), cysteinyl leukotrienes, platelet-derived growth factor (PDGF), epidermal growth factor (EGF), IL-6, and IL-1β, are essential for promoting ASM cell proliferation and differentiation [6,7,8,9,10,11]. Moreover, a crucial part of the pro-proliferative effect of eosinophil subtypes could be their direct adhesion through integrins on ASM cells or released extracellular matrix proteins. Integrins are transmembrane molecular mechanosensors that change their activation state in asthma, hereby not only regulating eosinophil adhesion, but transducing their activity and viability promoting signals via the cytoskeleton as well [12,13]. Eosinophils express seven transmembrane heterodimeric integrins, of which α_4_β_1_ and α_M_β_2_ are the most important in the context of asthma [6,14].

The pro-proliferative activity of eosinophil subtypes is unknown. We speculated that rEOS-like and iEOS-like cells could differ in their effects on the proliferation and apoptosis of ASM cells in asthma. Moreover, as we know that allergen-provoked acute asthma episodes could not equally affect the pro-proliferative properties of eosinophil subtypes [4], we hypothesized that a bronchial allergen challenge with *D. pteronyssinus* might result in accelerated eosinophil subtype-related development of airway remodeling during acute asthma. Lastly, eosinophil adhesion via integrins is essential for their functions. Hence, we sought to determine if the distinct eosinophil subtypes, rEOS-like and iEOS-like cells, could possess different integrin expression patterns.

## 2. Materials and Methods

The regional biomedical research ethics committee approved the study protocol for working with human subjects (BE-2-58). The study was registered in ClinicalTrial.gov with the identification number NCT04542902. All investigated individuals were introduced to the research protocols, and they confirmed their participation by signing the written informed consent form. Their data were depersonalized by assigning an appropriate number.

### 2.1. Study Design and Population

We included 16 patients with severe non-allergic eosinophilic asthma (SNEA) who were using high doses of inhaled steroids, 13 steroid-free patients with allergic asthma (AA), and 12 healthy nonsmoking subjects (HS) as a control group. Men and women aged 18–80 participated in the study. Patients were recruited from the Department of Pulmonology at the Hospital of Lithuanian University of Health Sciences Kaunas Clinics.

SNEA inclusion criteria were asthma diagnosed at least 12 months prior to the study, non-allergic phenotype; clinically confirmed and negative skin prick test; peripheral eosinophil count ≥ 0.3 × 10^9^/L at screening visit or ≥0.15 × 10^9^/L if eosinophil count ≥ 0.3 × 10^9^/L was recorded during the 12 months prior to sampling; no other reasons for the inadequate control of asthma; documented treatment of asthma with high doses of inhaled corticosteroids for at least 12 months in combination with a long-acting beta agonist ± a long-acting antimuscarinic drug ± episodic oral corticosteroids prior to enrollment; and two or more exacerbations of asthma that required treatment with systemic glucocorticoids during the 12 months prior to the scheduled visit.

AA inclusion criteria were newly diagnosed and untreated non-severe allergic asthma with symptoms and history longer than 12 months; a positive skin prick test to a clinically relevant allergen (*D. pteronyssinus*); and airway hyperresponsiveness during a methacholine challenge test.

HS inclusion criteria were no allergic or other chronic respiratory diseases; a negative methacholine challenge test; and a negative skin prick test.

Exclusion criteria for all the groups were clinically significant allergy symptoms; active airway infection one month prior to the study; exacerbation ≤ 1 month prior to the study; oral steroids ≤ 1 month prior to study; and smoking.

**Figure 1:**
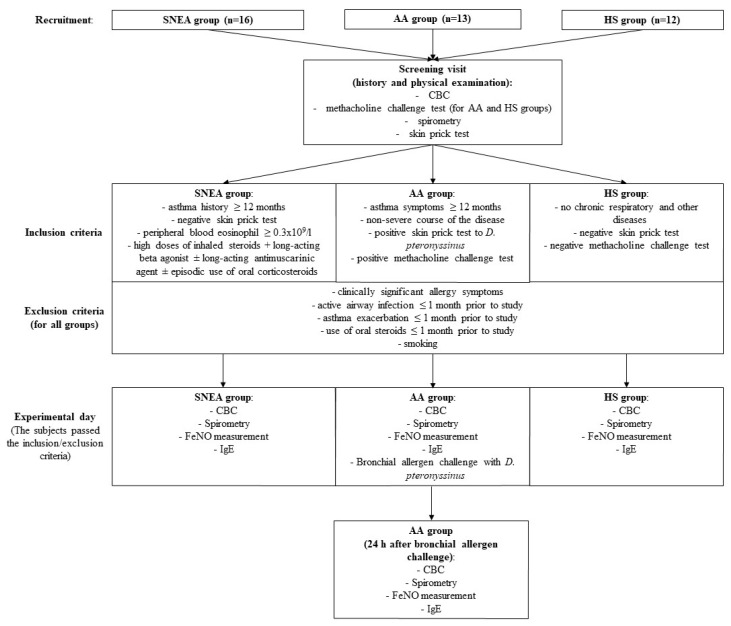
Study design and inclusion and exclusion criteria. AA, allergic asthma; CBC, complete blood count; FeNO, fractional exhaled nitric oxide; HS, healthy subjects; SNEA, severe non-allergic eosinophilic asthma.

All subjects were invited to the study no later than two weeks after the approval of the inclusion or exclusion criteria. SNEA patients and HS visited the clinic once and AA patients twice (at baseline and 24 h after the bronchial allergen challenge). During the first visit, complete blood count, spirometry, fractional exhaled nitric oxide (FeNO), and blood IgE levels were measured, and peripheral blood was collected from all study subjects. In addition, the bronchial challenge with the *D. pteronyssinus* allergen was performed on AA patients after peripheral blood had been drawn. The study design and inclusion and exclusion criteria are shown in Figure 1.

### 2.2. Experiment Plan

The granulocyte fraction was isolated from peripheral blood. During the initial quality control of the purification process, isolated granulocytes were counted. Their viability was determined; samples with greater than 98% granulocyte viability were considered to have passed quality control and were further used for eosinophil purification. The second quality control of the cell separation process was performed on the isolated eosinophils by counting and assessing their viability and purity by flow cytometry (with forward and side light scattering). Appropriate samples (>1.5 × 10^6^ cells/20 mL blood; viability > 98%, purity > 96%) were further used for the separation of eosinophil subtypes. The third quality control check measured the suitability of the collected rEOS-like and iEOS-like cells for further investigations: >0.5 × 10^6^ cells, viability > 97%.

**Figure 2:**
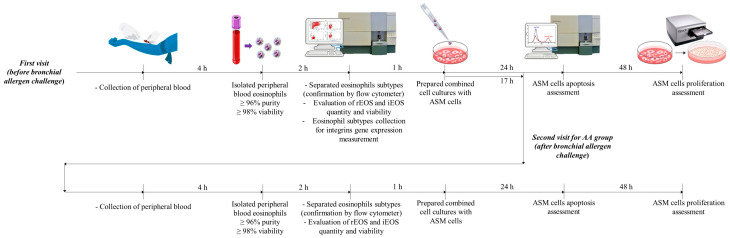
Experiment plan. AA, allergic asthma; ASM, airway smooth muscle; iEOS, inflammatory eosinophils; rEOS, lung-resident eosinophils.

After eosinophil subtyping, combined cell cultures with healthy immortalized ASM cells were immediately prepared, and their viability and proliferative properties were tested after 24 and 72 h of incubation, respectively. After purification of eosinophil subtypes, cells were frozen at −80 °C, and integrin gene expression measurements were performed after a sufficient number of eosinophil cells had been collected. The second AA appointment was 24 h after the bronchial allergen challenge, and all procedures except the eosinophil integrin gene-expression assessment were repeated according to the baseline. The experiment plan is shown in Figure 2.

### 2.3. Lung Function Testing

An ultrasonic spirometer was used to test the lung function (Ganshorn Medizin Electronic, Niederlauer, Germany). The results of forced expiratory volume in 1 s (FEV_1_), forced vital capacity (FVC), and the FEV_1_/FVC ratio were considered the largest of the three independent measurements, as described in [4].

### 2.4. Methacholine Challenge Test

A methacholine challenge test was performed using a pressure dosimeter (ProvoX, Ganshorn Medizin Electronic, Niederlauer, Germany) to detect airway hyperresponsiveness. Aerosolized methacholine was inhaled at 2 min intervals with a dose starting at 0.0101 mg that was gradually increased to 0.121, 0.511, and 1.31 mg until the total cumulative dose was achieved or a 20% decrease in FEV1 from the baseline was achieved. The bronchoconstriction effect of each methacholine dose was expressed as described in [4].

### 2.5. Skin Prick Testing

All patients underwent skin prick allergy testing with standardized allergen extracts (Stallergenes, S.A., Antony, France) for the following allergens: *D. pteronyssinus*, *D. farinae*, cat and dog dander, five mixed grass pollens, birch pollen, mugwort, *Alternaria*, *Aspergillus*, and *Cladosporium*. Diluent (saline) was used as a negative control and histamine hydrochloride (10 mg/mL) as a positive control. The skin prick test was read after 15 min of application. Skin prick test results were considered to be positive if the mean wheal diameter was greater than 3 mm. All AA patients were sensitized to *D. pteronyssinus*.

### 2.6. FeNO Measurement

Fractional exhaled nitric oxide (FeNO) analysis was performed on all study participants through an online method using a single exhalation and electrochemical assay (NIOX VERO, Circassia, UK) according to the methodology described in [4].

### 2.7. Bronchial Allergen Challenge Test

The *D. pteronyssinus* allergen (DIATER, Madrid, Spain) was inhaled via a pressure dosimeter (ProvoX, Ganshorn Medizin Electronic, Niederlauer, Germany). The starting point for the evaluation of the bronchoconstrictive effect was 2 min after inhalation of nebulized saline. The aerosolized allergen was inhaled at 10 min intervals, starting with an allergen concentration of 0.1 histamine equivalent (HEP)/mL. The whole procedure is described in [4].

### 2.8. Peripheral Blood Cell Analysis

Peripheral blood from each study subject was collected in vacutainers with dipotassium ethylenediaminetetraacetic acid (K2EDTA) (BD Vacutainer^®^, Becton Dickinson UK Ltd., Wokingham, UK). A UniCel^®^ DxH 800 Coulter^®^ Cellular Analysis System automated hematology analyzer (Beckman Coulter, Miami, FL, USA) was used for the complete blood count test.

### 2.9. Isolation of Eosinophils from Peripheral Blood and Eosinophil Subtyping

Approximately 24 mL of peripheral blood from each subject was delivered to the laboratory K2EDTA vacutainers, transferred to a tube, and diluted up to 50 mL with 1× phosphate-buffered saline (PBS) (GIBCO, Paisley, UK). Density gradient centrifugation was performed using Ficoll-Paque PLUS (GE Healthcare, Helsinki, Finland) as the whole blood was layered on Ficoll-Paque reagent and centrifuged at 300× *g* force for 30 min at room temperature. The supernatant was removed, and the layer of granulocytes and erythrocytes remained at the bottom. To remove erythrocytes, we used hypotonic lysis of erythrocytes by adding half the volume of sterile deionized water, gently mixing for up to 10 s, and immediately adding an equal volume of 2× concentrated PBS centrifuged at 300× *g* force for 10 min. The procedure was repeated until no red blood cells remained. Isolated granulocytes were counted, and the viability test was evaluated using an ADAM automatic cell counter (Witec AG, Sursee, Switzerland). Eosinophil enrichment was performed via magnetic activated cell sorting (MACS). Granulocytes were resuspended in cold MACS buffer (containing PBS (pH = 7.2), 0.5% bovine serum albumin (BSA), and 2 mM of EDTA) prepared by diluting MACS BSA Stock Solution 1:20 with autoMACS Rinsing Solution (40 μL for 10^7^ of all cells). The granulocyte suspension was incubated using the Eosinophil Isolation Kit (Human, MACS, Miltenyi Biotec, Somerville, MA, USA). The first incubation was performed by adding Biotin-Antibody Cocktail (biotin-conjugated monoclonal antibodies against CD2, CD14, CD16, CD19, CD56, CD123, and CD235A (glycophorin A) (10 μL for 10^7^ of all cells) to the granulocyte suspension for 10 min at 4 °C. The second incubation was performed for 15 min at 4 °C with the addition of Anti-Biotin MicroBeads (anti-biotin monoclonal antibodies, isotype mouse immunoglobulin G1 (IgG1) (20 μL for 10^7^ total cells)). During incubation, all cells except eosinophils were labeled with magnetic beads. The manufacturer certified that the eosinophil separation kit does not affect the viability of eosinophils and that the separation efficiency is greater than 96%. After incubation, eosinophils were separated by magnetic separation. LS columns (Miltenyi Biotec, Somerville, MA, USA) were placed in the MACS separation magnetic field stand (MACS Multistand, Miltenyi Biotec, Somerville, MA, USA). The column filter (30 μm, Miltenyi Biotec, Somerville, MA, USA) was rinsed with MACS buffer. The prepared cell suspension was applied to the pre-separation filter/LS column, and magnetically labeled cells remained on the LS column. Eosinophils flowed through the column into the tube. The separated eosinophil suspension was centrifuged at 300× *g* for 10 min at 22 °C. After centrifugation, the eosinophil count and viability were assessed using an ADAM automated cell counter (Witec AB, Sursee, Germany).

Eosinophil subtyping was performed using the magnetic beads’ conjugated antibodies (Miltenyi Biotec, Somerville, MA, USA) against CD62L, expressed on rEOS-like cell surfaces, but not on the iEOS-like cells [3]. The suspension of eosinophils was centrifugated at 300× *g* force for 10 min at room temperature, and the supernatant was completely aspirated and resuspended up to 10^7^ total cells per 60 µL of MACS buffer; 10 μL of FcR Blocking Reagent (Miltenyi Biotec, Somerville, MA, USA) per 10⁷ total cells was added, mixed well, and incubated for 10 min at 4 °C, and 20 μL of CD62L MicroBeads (Miltenyi Biotec, Somerville, MA, USA) were then added, resuspended, and incubated for 15 min at 4 °C. The cells were washed with an additional 2 mL of MACS buffer, centrifuged at 300× *g* force for 10 min at room temperature, and resuspended in 500 μL of the buffer. The cell suspension was loaded on a new LS column. All that passed through the column were iEOS-like cells, because they were unlabeled with CD62L, and eosinophils trapped in the column were rEOS-like cells. Labeled rEOS-like cells were collected by removing the LS column from the magnetic field and adding 500 μL of buffer to the column. The manufacturer declares that positive separation uses up to 10% of the selected surface proteins, and does not affect the activity of eosinophils. Both tubes with iEOS-like and rEOS-like cells were centrifuged at 300× *g* force for 10 min and counted, and their viability was assessed using an ADAM automated cell counter.

**Figure 3:**
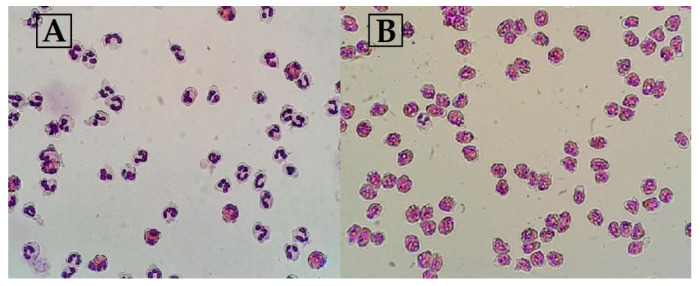
Eosinophil purity inspection by light microscopy after May–Grünward Giemsa staining. (**A**) Granulocytes; (**B**) eosinophils after magnetic separation.

Each time, quality control was ensured with flow cytometer FacsCalibur (BD, Franklin Lakes, New Jersey, USA), and the forward and side scattering were recorded, as eosinophils from granulocytes and eosinophil’ subtypes were distinguished by their granularity. Moreover, to examine eosinophil purity, the slides were prepared at each eosinophil isolation step using Thermo Scientific Cytospin 4 Centrifuge (Shandon Southern Instruments, Sewickley, PA, USA). Later, the prepared slides were stained using a UniCel^®^ DxH Slidemaker (Beckman Coulter, Miami, FL, USA) system with May–Grünwald Giemsa staining following the manufacturer’s protocol, and inspection by light microscopy was performed (Figure 3).

### 2.10. Combined Cell Cultures between Isolated Eosinophil Subtypes and Airway Smooth Muscle Cells

Individual combined cell cultures (co-cultures) of eosinophil subtypes and healthy human ASM cells were prepared for all experiments. ASM cells were immortalized by the stable expression of human telomerase reverse transcriptase (hTERT) as described in [15]. The same hTERT ASM cell line was used for all experiments with periodical renewal, avoiding ASM cell viability and activity changes. ASM cell lines were cultivated on plastic dishes in Dulbecco’s Modified Eagle’s Medium (DMEM; GIBCO by Life Technologies, UK) supplemented with streptomycin/penicillin (2% *v*/*v*; Pen-Strep, GIBCO by Life Technologies, Paisley, UK), amphotericin B (1% *v*/*v*; GIBCO, Paisley, UK), and fetal bovine serum (10% *v*/*v*; GIBCO by Life Technologies) under standard culture conditions of 5% CO_2_ in air at 37 °C, with medium renewal every three days. The preparation of ASM cells for assays in combined cultures with eosinophil subtypes was as previously described [4]. The ratio of ASM cells/eosinophils in the combined cell ratio was 4:1.

### 2.11. Airway Smooth Muscle Cell Proliferation Assay

For ASM cell proliferation measurements, cells were grown in 24-well plates under the conditions described above in a fetal bovine serum-supplemented growth medium until approximately 5 × 10^4^ cells/well confluence was reached. At 24 h before the experiments, the culture medium was changed to a serum-free medium. ASM cells were co-cultured for 72 h with an appropriate population of eosinophils isolated from SNEA, AA, or HS patients. All cells were then washed twice with 37 °C PBS, and the plates were gently tapped in the middle to remove residual eosinophils and again washed twice with warm PBS. Eosinophils are significantly less adherent than ASM cells, and can be mechanically removed; however, residual eosinophils did not significantly impact the final results due to their lower metabolic activity.

ASM cell proliferation was assessed by incubating the wells with Hank’s balanced salt solution containing Alamar blue (10% *v*/*v*; Invitrogen by Life Technologies, Paisley, UK). The conversion of Alamar blue into a reduced form is dependent on the metabolic activity of ASM cells. The conversion was evaluated by dual-wavelength spectrophotometry at wavelengths of 570 and 600 nm. According to the manufacturer, the degree of Alamar blue conversion is proportional to the number of viable cells in the culture.

Data are expressed as the percentage increase or decrease in Alamar blue conversion by ASM cells compared with that in the control cells (without co-culturing with eosinophil subtypes) that did not proliferate during the culturing period due to the usage of a serum-free medium. Alamar blue conversion was calculated based on Equation (1). The number of added eosinophils was 1.25 × 10^4^. The blood serum volume of the subjects was 2%.

(1) $$\mathrm{Percentage}\;\mathrm{difference}\;\mathrm{between}\;\mathrm{treated}\;\mathrm{and}\;\mathrm{control}\;\mathrm{ASM}\;\mathrm{cells}\;=\;\frac{\left(O2\times A1\right)-\left(O1\times A2\right)}{\left(R1\times N2\right)-\left(R2\times N1\right)}\;\times \;100$$

where O1 is a molar extinction coefficient of oxidized Alamar blue at 570 nm; O2 is a molar extinction coefficient of oxidized Alamar blue at 600 nm; R1 is a molar extinction coefficient of reduced Alamar blue at 570 nm; R2 is a molar extinction coefficient of reduced Alamar blue at 600 nm; A1 is the absorbance value of test wells at 570 nm; A2 is the absorbance value of test wells at 600 nm; N1 is the absorbance value of the negative control well at 570 nm; and N2 is the absorbance value of the negative control well at 600 nm.

### 2.12. Airway Smooth Muscle Cell Viability Assay

ASM cells were grown in six-well plates to a confluence of approximately 2 × 10^5^ cells/well. On the day of an experiment, a co-culture was prepared with 0.5 × 10^5^ of isolated eosinophils in a serum-free growth medium or a medium supplemented with 2% (*v*/*v*) of the subject’s blood serum. After 24 h of co-culturing, the plates were gently tapped in the middle to remove residual eosinophils. ASM cells were then trypsinized, collected into 1.5 mL tubes (Invitrogen, Life Technologies, UK), and centrifuged at 400× *g* for 10 min.

The fluorescein isothiocyanate (FITC) Annexin V Apoptosis Detection Kit II (BD Bioscience, San Jose, CA, USA) was used to assess cell viability, and the method was adapted according to the manufacturer’s recommendation. The viability of ASM cells was evaluated by fluorescent staining with annexin V for apoptotic cells and propidium iodide (PI) for necrotic cells. In addition, the controls of unstained cells, cells stained with FITC Annexin V, and cells stained with PI were used for each experiment. The viability of ASM cells that had not been co-cultured with eosinophils was determined as a control. Cell debris was excluded after the appropriate gating by forward and side scatter (FSC/SSC).

### 2.13. Gene Expression Assessment

The total RNA of eosinophil subtypes was extracted using a miRNeasy Mini Kit (Qiagen, Valencia, CA, USA) according to the manufacturer’s instructions. The expressions of the αM-, β1-, α4-, and β2-integrin subunit genes were determined for both eosinophil subtypes by qPCR using the commercial Power SYBR^®^ Green RNA-to-CT™ 1-Step kit (Applied Biosystems, Foster City, CA, USA) according to the manufacturer’s protocol. The process was performed for 40 repetitive cycles using the 7500 Fast Real-Time PCR system as follows: reverse transcription at 48 °C (30 min); activation of AmpliTaq Gold^®^ DNA polymerase, UP (Ultra-Pure) at 95 °C (10 min); denaturation at 95 °C (15 s); and annealing and extension at 60 °C (1 min). The obtained data were analyzed using the comparative cycle threshold method.

Primers used for gene expression analysis are shown in Table 1. The endogenous 18S ribosomal RNA gene concentration did not change in different samples; therefore, the expression of this gene was used as a housekeeping gene to normalize the data. Data are represented as logarithm-transformed fold changes.

### 2.14. Statistical Analysis

Statistical analysis was performed using GraphPad Prism 6 for Windows (ver. 9.1.1, 2021 GraphPad Software Inc., San Diego, CA, USA). The Shapiro–Wilk test was used to confirm the normality assumption of data distribution. The data distribution did not pass the normality test, so nonparametric tests were used. For multiple comparison analysis, the Kruskal–Wallis test was used; if it passed, the Mann–Whitney two-sided U-test was used for two independent groups when comparing the different effects of eosinophil subtypes on ASM cell proliferation and viability, as well as to compare distinct integrin subunit expression between different investigated groups; the Wilcoxon matched-pair, signed-rank, two-sided test was used for dependent groups when comparing eosinophil subtype differences from one patient. The minimal limit for statistically significant values was *p* < 0.05.

## 3. Results

### 3.1. Study Subject Characteristics

We investigated 41 individuals: 16 severe non-allergic eosinophilic asthma (SNEA) patients with high doses of inhaled steroids, 13 steroid-free allergic asthma (AA) patients, and 12 healthy subjects (HS) as a control group (Table 2). Men and women aged 18–80 participated in the study. SNEA patients were significantly older than those of the other groups. In addition, SNEA patients were characterized by a significant worsening of lung function and the highest blood eosinophil count compared to those of the AA and HS groups. FeNO was equally elevated in both AA and SNEA groups and higher in these groups compared to HS. IgE levels were significantly increased in AA and SNEA patients, with the highest value in the AA group.

The bronchial allergen challenge with *D. pteronyssinus* was performed for all AA patients, and significant early asthmatic responses were received with ≥20% fall in FEV_1_ from the baseline during the challenge. AA patients were re-evaluated after 24 h of the bronchial allergen challenge. A significant increase in blood eosinophils count to 0.53 (0.14–1.00) × 10^9^/L was received (*p* < 0.05). However, no significant differences were found in IgE and FeNO indicators after 24 h of the bronchial allergen challenge.

### 3.2. Eosinophil Subtype Integrin Expression Assessment

The adhesion of eosinophils occurs when their surface integrins recognize and bind to ASM cell adhesion molecules or to ligands on ASM cell-released extracellular matrix proteins. We previously demonstrated that the adhesion properties of eosinophil subtypes are closely related to the expression of eosinophil integrins [16]; thus, in this study, we investigated changes in the gene expression of α_4_β_1_ and α_M_β_2_ integrins in distinct eosinophil subtypes.

**Figure 4:**
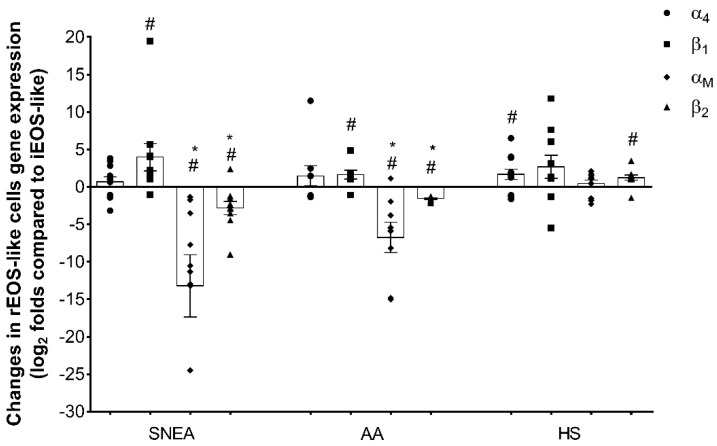
Gene expression of eosinophil subtypes, α_4_β_1_ and α_M_β_2_ integrins. SNEA, severe non-allergic eosinophilic asthma; AA, allergic asthma; HS, healthy subjects. AA patients, *n* = 9; SNEA patients, *n* = 11; HS, *n* = 12. * *p* < 0.05 compared with the HS group; # *p* < 0.05 compared with iEOS. Results are presented as the mean ± standard error. Statistical analysis between investigated groups, Mann–Whitney two-sided U-test; within one study group, comparing each point of the subject individually, Wilcoxon matched-pair, signed-rank, two-sided test.

Based on the qPCR analysis, rEOS-like cells isolated from both asthma groups showed a higher expression of the β_1_-integrin subunit gene: 4.0 ± 1.8-fold in the SNEA group and 1.6 ± 0.6-fold in the AA group (*p* < 0.05) compared to the expression of iEOS-like cells. No significant changes in the HS groups were found. Meanwhile, rEOS-like cells isolated from HS were characterized by the higher gene expression of α_4_- and β_2_-integrin subunits: α_4_, 1.7 ± 0.7-fold, and β_2_, 1.2 ± 0.3-fold compared to the expression of iEOS-like cells (*p* < 0.05). Moreover, iEOS-like cells from the SNEA and AA patient groups could be distinguished based on their significantly higher α_M_ and β_2_ integrin subunit gene expression over the rEOS-like cells: in the SNEA group, α_M_ increased by 13.2 ± 4.2-fold; in β_2_, by 2.8 ± 0.9-fold (*p* < 0.05). In the AA group, α_M_ increased by 6.7 ± 2.0-fold; β_2_, by 1.6 ± 0.1-fold (*p* < 0.05). In both asthma groups, the increase in gene expression of α_M_ and β_2_ integrin subunits was higher compared with the iEOS-like cells of the HS group (*p* < 0.005; Figure 4).

### 3.3. Eosinophil Subtype Effect on Airway Smooth Muscle Cell Proliferation

Asthmatic eosinophils have a more pronounced pro-proliferative effect on ASM cells [16]. In this case, we investigated the pro-proliferative properties of distinct eosinophil subtypes. Our results showed that, after 72 h in co-cultures with ASM cells, both eosinophil subtypes had significantly increased proliferation of ASM cells compared to control ASM cells that had not co-incubated with eosinophils (*p* < 0.05). However, rEOS-like cells had higher pro-proliferative properties compared to iEOS-like cells in all studied groups (*p* < 0.05).

**Figure 5:**
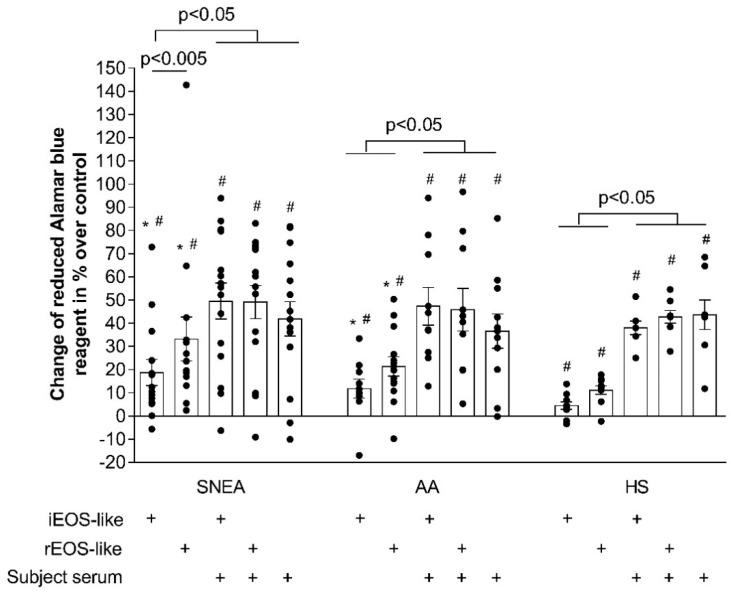
ASM co-culture with either rEOS-like or iEOS-like cells and the effect on ASM cell proliferation. SNEA, severe non-allergic eosinophilic asthma; AA, allergic asthma; HS, healthy subjects. Results from independent experiments of SNEA, *n* = 16, AA *n* = 13, HS *n* = 12. ^#^ *p* < 0.05 compared with control ASM cells not co-cultured with eosinophils; * *p* < 0.05 compared with the HS group. The final concentration of the added blood serum was 2% *v*/*v*. Statistical analysis: between investigated groups, Mann–Whitney two-sided U-test; within one study group, comparing each point of the subject individually, Wilcoxon matched-pair, signed-rank, two-sided test.

In the SNEA group, the ASM cell number in the co-culture with rEOS-like cells was increased by 33.2% ± 9.4%, and by 18.7% ± 5.6% with iEOS, compared to ASM cells that had not been co-incubated with eosinophils (*p* < 0.05). Similarly, in the AA group, the inclusion of rEOS-like cells promoted ASM cell proliferation by 21.4% ± 4.1% and by 11.7% ± 4.1% with iEOS-like cells (*p* < 0.05). In the HS group, the inclusion of rEOS-like cells enhanced ASM cell proliferation by 11.1% ± 1.8%, and by 4.5% ± 1.6% with iEOS-like cells (*p* < 0.05). Moreover, the effect of co-culture with rEOS-like and iEOS-like cells on ASM cell proliferation did not differ between asthma groups, but was significantly stronger in comparison to co-cultures with the HS group (*p* < 0.05; Figure 5).

Supplementation of the growth medium with 2% of the subject’s serum was found to increase ASM cell proliferation in all studied groups compared with control ASM cells incubated in a medium that had not been serum-supplemented (*p* < 0.05). In the SNEA group, proliferation increased by 41.9% ± 7.4% when only ASM cells were cultured in the serum-supplemented medium (*p* < 0.05), and the results did not differ when ASM cells were cultured in the serum-supplemented medium together with rEOS-like or iEOS-like cells (proliferation increased by 49.1% ± 7.2% and 49.5% ± 7.7%, respectively; *p* < 0.05). We obtained similar results in the AA group (the proliferation of ASM cells increased by 36.6% ± 7.4% when ASM cells were cultured with serum-supplemented medium only, by 45.9% ± 9.2% with rEOS-like cells; *p* < 0.05, and by 47.3% ± 8.1% with iEOS-like cells; *p* < 0.05), and in the HS group (proliferation increased by 43.6% ± 6.3%; *p* < 0.05, by 42.7% ± 2.8%; *p* < 0.05, and by 38.0% ± 3.0%; *p* < 0.05, respectively; Figure 5).

### 3.4. Effect of Allergen-Activated Eosinophil Subtypes on Airway Smooth Muscle Cell Proliferation

The effect of an in vivo provoked acute allergic asthma episode after the bronchial allergen challenge to eosinophil subtypes’ pro-proliferative properties was determined by comparing the results before and 24 h after the bronchial allergen challenge of the same subject.

**Figure 6:**
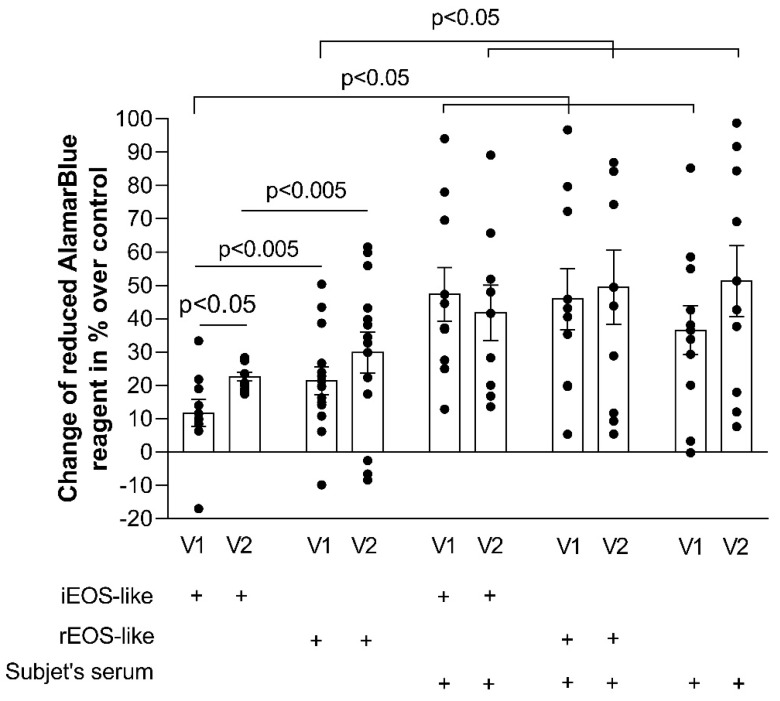
Effect of the bronchial allergen challenge on rEOS-like and iEOS-like cells promoted ASM cell proliferation. AA, allergic asthma; V1, visit 1 (before the bronchial allergen challenge); V2, visit 2 (24 h after the bronchial allergen challenge). Results from independent experiments of AA, *n* = 13. Statistical analysis: between investigated groups, Mann–Whitney two-sided U-test; within one study group, comparing each point of the subject individually, Wilcoxon matched-pair, signed-rank, two-sided test.

Our results showed that the bronchial allergen challenge only significantly enhanced the iEOS-like cells’ effect on ASM cell proliferation. The ASM cell number after 72 h of incubation in a co-culture with allergen-activated iEOS-like cells increased by 22.5% ± 1.3% compared to that of ASM cells without incubation with eosinophils (*p* < 0.005), and was significantly greater than the effect of allergen non-activated iEOS-like cells (*p* < 0.05). The bronchial allergen challenge had no significance on the pro-proliferative properties of rEOS-like cells. Although the effect of iEOS-like cells was enhanced after the bronchial allergen challenge, rEOS-like cells still had a stronger impact, as the ASM cell number increased by 29.8% ± 6.2% (*p* < 0.005). Furthermore, the addition of blood serum collected 24 h after the bronchial allergen challenge to the medium had no additional pro-proliferative effect on ASM cell proliferation compared to blood serum collected before the bronchial allergen challenge, and it had no additional eosinophil-activating effect (Figure 6).

### 3.5. Eosinophil Subtype Effect on Airway Smooth Muscle Cell Apoptosis

**Figure 7:**
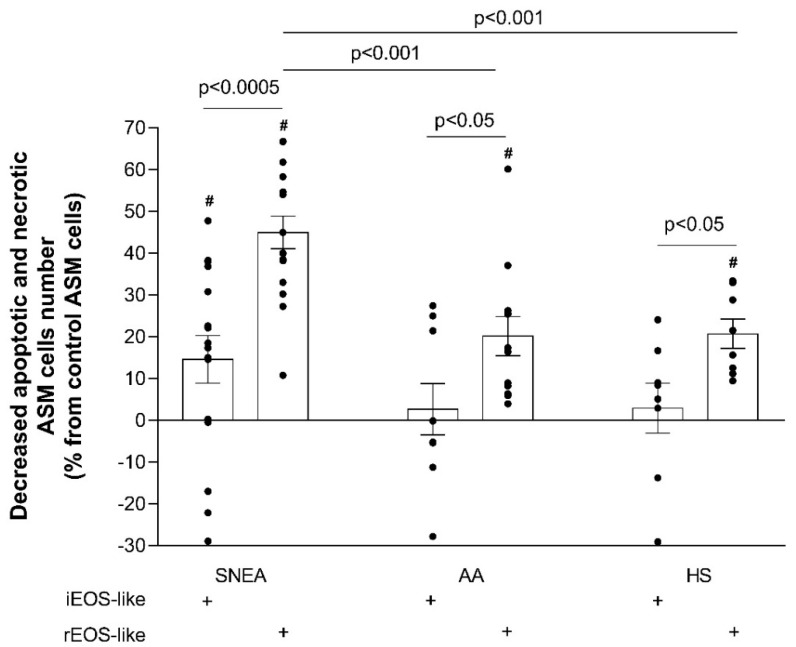
rEOS-like and iEOS-like cells’ effect on ASM cell apoptosis. SNEA, severe non-allergic eosinophilic asthma; AA, allergic asthma; HS, healthy subjects. Results from independent experiments of SNEA, *n* = 16, AA *n* = 13, HS *n* = 8. ^#^ *p* < 0.05 compared with control ASM cells without co-culturing with eosinophils; *p* < 0.05 compared with the HS group. Statistical analysis: between investigated groups, Mann–Whitney two-sided U-test; within one study group, comparing each point of the subject individually, Wilcoxon matched-pair, signed-rank, two-sided test.

We determined the effect of eosinophil subtypes on the apoptosis of ASM cells after 24 h of co-culture and presented the results in comparison with those of control ASM cells without incubation with eosinophils; rEOS-like cells isolated from SNEA patients had a more pronounced effect in reducing ASM cell apoptosis compared to the AA and HS groups: the number of apoptotic ASM cells decreased by 45.0% ± 3.9%, 20.1% ± 4.7%, and 20.7% ± 3.5%, respectively (*p* < 0.001). Moreover, the rEOS-like cells’ effect was stronger than that of iEOS-like cells in all studied groups (*p* < 0.05); iEOS-like cells only significantly reduced ASM cell apoptosis in the SNEA group (the number of apoptotic ASM cells was decreased by 14.6% ± 5.7%; *p* < 0.05; Figure 7).

### 3.6. Effect of Allergen-Activated Eosinophil Subtypes on Airway Smooth Muscle Cell Apoptosis

**Figure 8:**
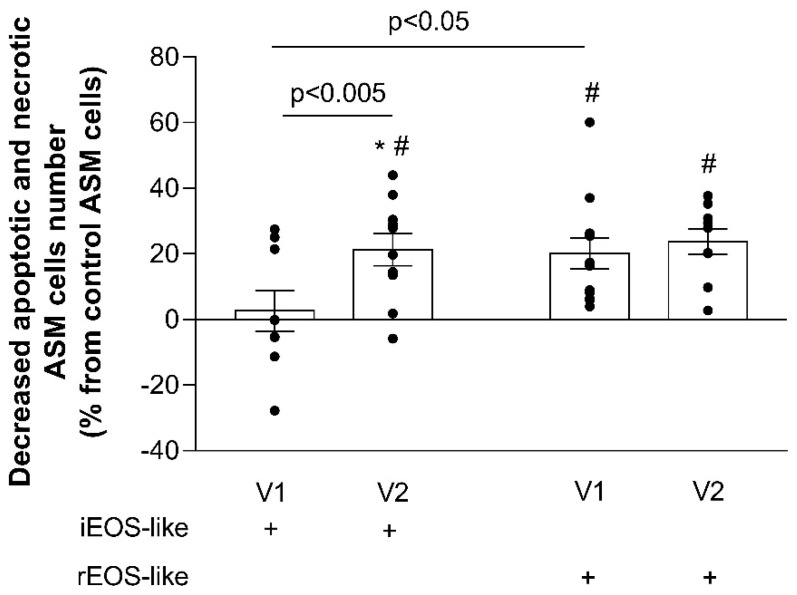
Effect of the bronchial allergen challenge on rEOS-like and iEOS-like cells promoted ASM cell apoptosis. AA, allergic asthma; V1, visit 1 (before the bronchial allergen challenge); V2, visit 2 (24 h after the bronchial allergen challenge). Results from independent experiments of AA, *n* = 13. ^#^ *p* < 0.05 compared with control ASM cells without co-culturing with eosinophils; * *p* < 0.05 compared with the HS group. Statistical analysis: between investigated groups, Mann–Whitney two-sided U-test; within one study group, comparing each point of the subject individually, Wilcoxon matched-pair, signed-rank, two-sided test.

Lastly, following the promotion of acute asthma episodes through the bronchial allergen challenge, it was found that the effect of iEOS-like cells on the reduction in ASM cell apoptosis was enhanced. After interaction with activated iEOS-like cells, the number of apoptotic ASM cells in the culture decreased by 23.8% ± 3.9% (*p* < 0.005), and was significantly increased compared to that of non-activated eosinophils (*p* < 0.005). The bronchial allergen challenge had no additional effect on rEOS-like cells. After the bronchial allergen challenge, the effects of rEOS-like and iEOS-like cells on the reduction in ASM cell apoptosis became equal (Figure 8).

## 4. Discussion

Blood rEOS-like and iEOS-like cells demonstrated a pro-proliferative effect on ASM cells through co-culture, and this effect of asthmatic cells exceeded that of healthy ones; rEOS-like cells had stronger pro-proliferative properties compared with that of iEOS-like cells, however, the bronchial challenge with the *D. pteronyssinus* allergen significantly enhanced the pro-proliferative properties of iEOS-like cells without affecting rEOS-like cells in AA patients. Lastly, rEOS-like cells isolated from SNEA and AA patients’ blood possessed enhanced expression of the β_1_ integrin subunit, while blood iEOS-like cells had both α_M_β_2_ integrin subunits.

Tissue eosinophils maintain homeostasis in steady state conditions; however, they play an important role in host defense against viral, parasitic, fungal, and bacterial infections through eosinophil-derived cytotoxic mediators packed in their granules [17,18,19,20]. Eosinophils are a hallmark of airway inflammation in asthma, and may contribute to chronic airway hyper-responsiveness due to active contribution to ASM cell proliferation, leading to increased ASM mass [16,21]. An increased blood eosinophil count could be associated with further infiltration into the airways, and due to the abundant release of eosinophil-derived mediators, they are associated with detrimental effects in airway inflammation. However, the blood eosinophil count without focusing on the predominant eosinophil subtype could not sufficiently reflect their role and count in the airways. Eosinophil subtypes differ by their biological properties: rEOS express genes related to immune response regulation and tissue homeostasis, while iEOS have a high expression of pro-inflammatory genes [3]. These cells are classified according to morphological changes, including differences in surface expression markers and density that could facilitate their distribution from the whole eosinophil count during the clinical investigation of asthmatic patients. On the basis of differences in surface expression markers, blood and tissue rEOS specifically express the surface molecule L-selectin, also known as CD62L [3]. Furthermore, on the basis of differences in granularity-related eosinophil subtype density, rEOS and iEOS were characterized by normodense and hypodense eosinophils, respectively [22]. Moreover, blood rEOS-like cells in inflammation conditions in the presence of pro-inflammatory mediators can demonstrate distinct functions compared to those in steady-state.

The current treatment of eosinophilia perspectives focuses on blood eosinophil depletion [23] or inhaled steroids that affect eosinophils in the lungs. We used a combined blood eosinophil and ASM cell culture model by simulating the processes in vivo. The usage of inhaled medications could not equally affect both eosinophil subtypes due to distinct localization in the lung tissue; therefore, the investigation of blood eosinophils as therapeutic targets could prevent their negative effect on the early stages before their primary effect on ASM cells.

Evidence shows that eosinophils contribute to ASM remodeling through direct contact via Th2 chemokine-activated integrin–ligand interaction [21,24]. Eosinophil surface integrins recognize and bind to ASM cell adhesion molecules and trigger signal transduction that controls cell growth, apoptosis, cellular differentiation, and division [25,26]. The intensity of adhesion is closely related to the expression of integrins and their activity stage. Infiltration of eosinophils from the blood into the airways, as well as their further biological function, also depends on these factors. The expression of α_4_β_1_ and α_M_β_2_ integrins in eosinophils from SNEA and AA was significantly higher compared with those from HS [27]. Primary selected asthma-related integrins of the eosinophil subtype analysis on a gene expression level could further focus on subtype separation on the basis of their biological functions; rEOS-like cells demonstrated more stable adhesion compared to that of iEOS-like cells [4]. Our study results revealed that stronger rEOS-like cell adhesion and thus prolonged pro-proliferative properties in asthma might be due to the increased expression of the β_1_ integrin subunit. This integrin interface with the α_4_ subunit is closely related to adhesion on the vascular cell adhesion molecule (VCAM)-1 in a P-selectin-dependent manner [28]. However, we did not obtain significant enhancement of the α_4_ integrin subunit, and could conclude that the expression of β_1_ is a limiting factor for this integrin heterodimer’s functions.

The activation of blood eosinophils with IL-5 results in increased expression of α_M_β_2_ and eosinophil adhesion to VCAM-1 and ICAM-1 via an α_M_β_2_-dependent mechanism [29,30]. The most distinct differences between eosinophil subtypes were the expressions of both α_M_β_2_ integrin subunit genes; iEOS-like cells are distinguished by much higher mRNA levels of this integrin compared to rEOS-like cells. However, Johansson et al. reported that IL-5, IL-3, or granulocyte macrophage colony-stimulating factor (GM-CSF) stimulates eosinophil adhesion to periostin through the α_M_β_2_ integrin [31]. In contrast to these results, these findings could be related to the blood iEOS-like cells population, which demonstrates the significant increase in α_M_β_2_ integrin expression. Moreover, iEOS attachment to periostin could both modulate their chemoattraction, transmigration, and adhesion [32] and act as an activator. These findings suggest that activation by periostin via α_M_β_2_ integrin could be an important feature for a more detailed understanding of iEOS functions in asthma. Taken together, evidence about distinct eosinophil subtype integrin expression is important for understanding their recruitment, activation, and further survivability in lung tissue. Controlling the suppression of eosinophil integrins could completely prevent eosinophil damage at the primary stage.

An important factor of airflow limitation in asthma is the degree of ASM remodeling, which includes ASM thickening due to increased ASM cell proliferation, hypertrophy, and extracellular matrix protein expression [33]. Asthmatic eosinophils significantly increase the proliferation of ASM cells compared with HS eosinophils, which may not even be related with eosinophil-derived mediators, but with their increased viability after adhesion on ASM cells or their released extracellular matrix proteins [7]. This was confirmed with the study of different eosinophils subtypes. It was observed that rEOS-like cells had higher adhesion intensity and were more sensitive to adhesion-related viability than iEOS-like cells [4,6]. The different eosinophil subtypes thus have a distinct effect on ASM cell proliferation and viability.

rEOS under physiological conditions regulate various important biological functions in the lung and prevent the development of T helper type 2 (Th2) immunity against inhaled allergens, thus contributing to the maintenance of lung homeostasis [3]; however, rEOS reflect some detrimental aspects. The inclusion of rEOS-like cells demonstrated a greater effect on ASM cell proliferation compared to iEOS-like cells in all the investigated groups. The enhanced activity of rEOS-like cells on ASM cell proliferation may be related to overexpressed homeostatic rEOS functions; rEOS showed remarkable ability for tissue repair and regeneration [22]; therefore, the constant attempt to ensure the stable regeneration of structural cells during asthma conditions may be associated with impaired pro-proliferative function. Lastly, rEOS could be associated with IL-4-driven regenerative responses to tissue injury [34,35] and with initiating efficient airway tissue regeneration involving the activation and proliferation of ASM cells.

iEOS are highly activated inflammatory cells that secrete large amounts of inflammatory mediators; however, iEOS-like cells their effect on ASM cell proliferation was significantly weaker than that of rEOS-like cells. The balance of homeostatic modeling and disease remodeling function of iEOS could be disturbed, and they cannot even induce structural cell proliferation, though it can be disturbed via the release of cytotoxic proteins. Primary iEOS functions are involved in branching morphogenesis [36], and they are invited into the lungs after the first breaths of newborns. This suggests that their effector functions could be shifted to the homeostatic modeling side during asthmatic conditions, thus inducing ASM cell proliferation instead of disruption.

The serum is a source of various cytokines, growth factors, and other biologically active mediators. Furthermore, increased levels of pro-inflammatory mediators are found in the blood of asthmatic patients. Our results demonstrated that blood serum is more important for ASM cell proliferation and has a higher proliferative effect compared with the iEOS-like cells of the SNEA group and with both eosinophil subtypes in the AA and HS groups. However, the pre-activation of isolated eosinophil subtypes with mediators found in the blood serum does not affect their pro-proliferative properties. This could mean that eosinophil subtypes do not lose their primary activity after 72 h of co-culture with ASM cells.

Exacerbations of asthma describe acute or semi-acute episodes of shortness of breath, wheezing, chest tightness, cough, or a combination of these symptoms. Exacerbation of the disease also affects quality of life, increases the risk of faster deterioration of lung function, and, in rare cases, could lead to death. After the bronchial allergen challenge, iEOS-like cells isolated from AA patients were activated; however, this had no significant effect on pro-proliferative rEOS-like cells properties; rEOS in inflammatory conditions are referred to as Type 1 eosinophils with a more segmented nucleus than that of steady state eosinophils [37]. These eosinophils are not actively involved in immune responses with basic functions to prevent the allergen-induced type 2 immunity; thus, the bronchial allergen challenge does not affect pro-proliferative rEOS-like cells functions. In contrast to rEOS-like cells, a recent study revealed that the peripheral blood iEOS-like cell level was reduced after dust mite-induced airway inflammation in the case of allergic asthma [4], possibly due to eosinophils’ migration to the airways after activation by mediators of type 2 airway inflammation.

Cell apoptosis is programmed cell death. The lack of apoptosis in ASM cells could be a component leading to uncontrolled proliferation [38,39] and further ASM hyperplasia [40]. Therefore, airway remodeling is closely related to the imbalance between the proliferation and apoptosis of ASM cells. Our results revealed that the rEOS-like cells of SNEA patients had a stronger impact on ASM cell apoptosis than those of the AA and HS groups, which may be related to the severe form of the disease and a more pronounced impaired rEOS-like cells effect. Apoptosis measurements also demonstrated that, during allergen-induced late-phase airway inflammation in the AA group, the effect of iEOS-like cells on the decrease in ASM cell apoptosis was enhanced. We used the most common *D. pteronyssinus* house dust mite allergen with which humans constantly come into contact. We did not perform the bronchial allergen challenge with the HS group, as this group was unsensitized to *D. pteronyssinus*. We previously described that inhaled high doses of concentrated allergen do not affect bronchoconstriction to HS, but are sufficient to stimulate type 2 inflammation and slightly activate iEOS [4]. This is important in future research to understand the possible development of AA later in life; however, research about this activation was not the purpose of the current study.

Asthma is a heterogeneous disease [41]; therefore, our in vitro co-culture model may not reflect the complex interactions with the tissue microenvironment in vivo. However, one of our research aims was to investigate the activation states of blood eosinophils. Therapies against blood eosinophils could prevent their primary effect before being suppressed by inhaled medications. Moreover, information that blood eosinophils are sufficiently activated to affect ASM cells in vitro suggests that our model is close to the in vivo processes [42,43]. However, the activation of eosinophil subtypes found in airways might be different. rEOS, due to their specific parenchymal localization, are less accessible by released type 2 inflammatory cells, especially during allergen-induced inflammation. Moreover, rEOS, unlike iEOS, are less affected by type 2 inflammatory mediators during allergen-induced inflammation due to their specific parenchymal localization. Due to this reason, results found in in vitro studies with blood eosinophil subtypes must also be confirmed by in vivo experiments with tissue eosinophils.

Our study has several limitations. In the assessment of ASM cell viability, annexin V^+^/PI^−^-stained cells were classified as early apoptotic, whereas annexin V^+^/PI^+^-stained cells were classified as late apoptotic or necrotic. However, annexin V staining may not necessarily indicate cell death only. Transient phosphatidylserine exposures may be due to lipid reconstitution, ATP depletion [44], or changes in cellular calcium concentrations [45]. To avoid as many inaccuracies as possible, control cells (ASM cells without co-culturing with eosinophils) were used to eliminate nonstandard conditions and normalize the results. Moreover, there is no information that eosinophils can induce nonspecific transient phosphatidylserine exposure by the mechanisms mentioned above. Our study of integrin gene expression also requires further investigation. The individuals’ blood serum samples were used for eosinophils pre-activation and could not disclose the unique effect on ASM cells. In order to define it, appropriate control serum supplements should be used. The currently obtained results are only at the gene expression level; consequently, it is necessary to assess the abundance of integrins formed on the surface of eosinophils in order to fully demonstrate the importance of changes in their expression in asthma. Another limitation was that evaluating allergen-activated eosinophils activity, early allergic responses were registered for all AA patients; however, the investigated individuals were not tracked for late allergic responses, which was not a study objective, but could potentially induce more intense eosinophilic Th2 inflammation. Moreover, not all isolated eosinophils remain viable after 72 h of incubation with ASM cells, and our proliferation data might be related to eosinophils’ survivability in a co-culture. However, we presume that activated eosinophil could rapidly release the long-acting pro-proliferative mediators. 

In conclusion, the relevance of the interaction between eosinophil subtypes and lung structural cells in the pathogenesis of asthma is constantly increasing, and should be thoroughly elucidated to allow the development of precise and effective individualized treatment. These findings demonstrate the different functional properties among eosinophil subtypes and highlight the importance of eosinophil subtype-orientated therapies targeting the development of airway remodeling in asthma.

## Figures and Tables

**Table 1:** Sequences of primers used for gene expression analysis.

Primer	Forward 5′–3′	Reverse 5′–3′
18S	5′-CGC CGC TAG AGG TGA AAT TC-3’	5′-TTG GCA AAT GCT TTC GCT C-3′
α_M_	5′-CAG ACA GGA AGT AGC AGC TCC T-3′	5′-CTG GTC ATG TTG ATG AAG GTG CT-3′
β_1_	5′-GTG TGG CCC AAG ACA GTT CT-3′	5′-GGT TAC CCC ACC CTC TGA CT-3′
α_4_	5′-GCT TCT CAG ATC TGC TCG TG-3′	5′-GTC ACT TCC AAC GAG GTT TG-3′
β_2_	5′-AAC GTA TGC GAG TGC CAT TC-3′	5′-TTC ACG GGG TTG TTC GAC AG-3′

**Table 2:** Demographic and clinical characteristics of the study population.

	SNEA Patients	AA Patients	Healthy Subjects
Number, *n*	16	13	12
Gender, M/F	3/13	9/4	5/7
Age, years	59.4 ± 2.8 *	28.9 ± 3.2 ^#^	33.6 ± 3.7
BMI, kg/m^2^	28.3 ± 1.3	23.8 ± 0.9	25.5 ± 1.3
PD_20M_, geometric mean (range), mg	ND	0.4 (0.12–0.95)	ND
PD_20A_, geometric mean (range), HEP/mL	ND	0.7 (0.08–2.13)	ND
IgE, geometric mean (range), IU/mL	63.1 (3.0–794.9) *	154.5 (13.9–940.0) *^,#^	17.8 (3.0–46.7)
FEV_1_, l	1.5 ± 0.17 *	3.8 ± 0.2 ^#^	4.0 ± 0.3
FEV_1_, % of predicted	53.9 ± 5.0 *	89.1 ± 2.4 ^#^	103.4 ± 2.4
Blood eosinophil count, geometric mean (range), ×10^9^/L	0.61 (0.06–2.20) *	0.46 (0.14–1.00) *^,#^	0.17 (0.06–0.50)
FeNO, geometric mean (range), ppb	38.2 (10.0–89.0) *	51.1 (18.0–135.0) *	14.4 (10.3–20.0)

AA, allergic asthma; F, female; FeNO, fractional exhaled nitric oxide; FEV_1_, forced expiratory volume in 1s; IgE, immunoglobulin E; M, male; ND, not done; PD_20A_, provocation dose of allergen causing a 20% decrease in FEV_1_; PD_20M_, provocation dose of methacholine causing a 20% decrease in FEV_1_; SNEA, severe non-allergic eosinophilic asthma. Data are presented as mean ± standard error of the mean, except PD_20M_ and PD_20A_, provided as a geometric mean (range). * *p* < 0.05 compared with the HS group; ^#^ *p* < 0.05 compared with the SNEA group. Statistical analysis, Mann–Whitney two-sided U-test.

## Data Availability

All the data presented in this study are included in this article.
